# Use of a large language model integrated within the electronic medical record for the evaluation of surgical site infections – Northern California, 2025

**DOI:** 10.1017/ice.2026.10432

**Published:** 2026-06

**Authors:** Eugenia Miranti, Timothy Keyes, Alvaro Ayala, Nerissa Ambers, Gina Newman, Elmer de Leon, Erika Paola Viana-Cardenas, Wajeeha Tariq, Mindy Sampson, Jorge L. Salinas

**Affiliations:** 1 Stanford Medicine Health Care, USA; 2 Stanford University School of Medicine, USA

## Abstract

Our study evaluated a large language model (gpt-4o-mini) for surgical site infection (SSI) adjudication, achieving 100% sensitivity but 69.4% specificity. While reducing the manual screening workload by 66%, the agent generated many false positives, underscoring the need for refined models to improve specificity without compromising accuracy.

## Introduction

Surgical site infections (SSIs) represent an important cause of morbidity, mortality, and increased health care costs. With more than 100,000 SSIs documented in annual prevalence surveys,^1^ they may increase mortality risk by as much as 11-fold, with cost of hospitalization increased by more than $20,000 per admission.^2^ Surveillance for SSIs can reduce risk, but the active, prospective chart review involved is labor-intensive, requiring an infection prevention professional to apply surveillance definitions as laid out in the National Health and Safety Network (NHSN) reporting guidelines.^3,4^

There is a potential role for large language models (LLMs) to reduce the clinical workload in SSI adjudication. LLMs have demonstrated impressive ability to answer medical board exam questions and make diagnoses; however, few studies of LLMs utilize real patient care data.^5^ We aimed to evaluate the accuracy of LLMs to query patients’ postsurgical notes for criteria supporting the NHSN’s SSI reporting guidelines.

## Methods

We audited 5,299 surgical cases from January to December 2023, reviewing patients’ postsurgical notes for criteria supporting the NHSN’s SSI reporting guidelines. Of these 5,299 patients, 146 patients had a postsurgical SSI detected via manual review (classic surveillance using NHSN definitions applied by an infection prevention professional for 28 procedures preselected using Epic Bugsy (Verona, WI), see supplement). We designated these “True Positives,” the gold standard to which the LLM would be compared. Informal debrief discussions were conducted virtually with Infection Preventionists (IPs) interfacing with the LLM during the pilot. IPs shared their experiences, lessons learned, and recommendations related to the use of LLMs for SSI adjudication.

**Figure 1.**
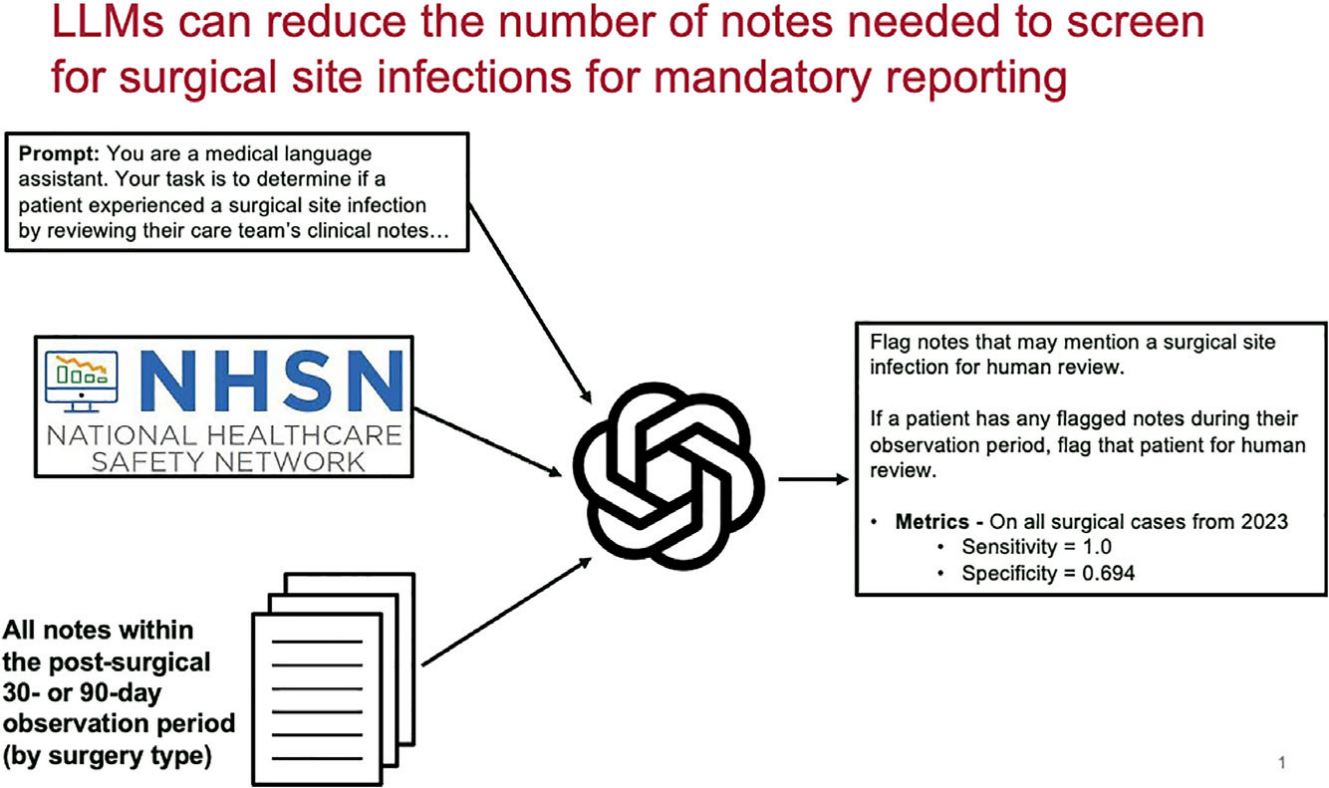
Figure 1 long description. Flow diagram of a large language model evaluation of surgical site infections, Northern California, 2025.

Stanford University has made secure LLM-based conversational applications with direct access to electronic medical records available for provider use. Using these tools, we developed a task-specific AI agent, *ChatEHR SSI solution* (gpt-4o-mini; OpenAI). After being prompted with the procedure name, the tool would scan patient notes (e.g., admission and progress notes, surgical notes) during the SSI surveillance period. The tool did not review laboratory or radiologic data, it only accessed notes. The tool was prompted to check for superficial SSI criteria, deep SSI criteria, and organ-space SSI criteria, using NHSN surveillance definitions^2^ (Figure 1). For each patient, the agents provided a response regarding the presence or absence of a SSI, the type (superficial, deep, or organ-space), and a brief supporting explanation. Notes that were identified as mentioning a SSI were flagged for review by an Infection Prevention professional. The prompts used can be found in Supplementary Material. A convenience sample of 50 random false positives across all procedures detected by the LLM was evaluated for common themes. Unstructured interviews of two Infection Prevention professionals were conducted by an expert in qualitative analyses (AGW) and theme AGW was performed. This study was approved by the Stanford Institutional Review Board.

## Results

The agent displayed 100% sensitivity, identifying all 146 True Positive SSIs initially identified by manual review. There were 0 False Negatives. Specificity was more modest at 69.4%, with the model flagging 1576 False Positive SSIs, which was excluded by the human reviewers (Figure 1). Additional metrics included 8.48% positive predictive value, and 100% negative predictive value. A random sample of 50 False Positive SSIs were reviewed, and three major patterns emerged among these over-calls: (1) misclassifying non-infectious postoperative fluid collections (i.e., seromas); (2) misclassifying “discharge” or “output”—even when characterized as *bloody* or *serous*; (3) misclassifying patients who underwent surgery to treat an *existing* infection.

In debriefs with two infection preventionist professionals, IP’s opinions reflected support with some reservations, including (1) the use of LLMs would take more time; (2) potential costs of using LLM; (3) that LLM’s utility was limited by the type of data used as part of its decision-making process. For example, the LLM did not include laboratory information, external data systems, or paper documents, which are critical elements to the IP decision-making process. IPs saw the LLM as an “extra set of eyes,” raising confidence that “a no is really a no.” IPs stated that lessons learned from this pilot include the importance of incorporating NHSN case definitions into the LLM algorithm, the need for IPs to receive education and training on LLMs, and ensuring IPs are part of the LLM development and implementation process from the very beginning. The latter two recommendations are particularly critical as the “adoption of new technology requires trust” and IPs need to understand how the LLM was developed and where data comes from. Overall, IPs did not perceive that the LLM would replace their expertise but would serve to augment their work, and that this could help expand the scope of SSIs included in surveillance efforts and could give IPs more time to be “out on the floor doing more boots on the ground IP work.”

## Discussion

In this study, AI agents demonstrated excellent sensitivity but limited specificity and precision in identifying SSIs, relative to infection prevention professional adjudication. These findings align with our prior work on LLMs and central line-associated bloodstream infections (CLABSI).^6^ In that study, the model (ChatGPT 4.0) was given two progress notes and blood culture results to determine whether the NHSN CLABSI criteria were met. Without fine-tuning, retrieval augmentation, or few-shot prompting, it achieved 80% sensitivity and 35% specificity. When missing clinical information was added, sensitivity and specificity improved to 90% and 75%, respectively. This supports a role for LLM use in adjudication tasks that involve criteria matching within precise NHSN surveillance definitions.

For the current tool, we considered one utility to be the potential reduction in human workload without compromise of accuracy in facilities with medical record systems that do not include a function to identify patients with potential SSIs. This aim was achieved, as our tool resulted in a reduction in the number of patients needed for manual screen from 5,299 (all patients) to 1722 (only the patients flagged by the LLM). Thus, our tool produced a 66% reduction in manual screening workload (in terms of number of notes needed to review) without any additional risk of “missing” patients with SSIs. This practical benefit underscores the potential of LLMs to significantly alleviate the burden on infection prevention professionals involved in labor-intensive chart reviews for SSIs, even if human intervention remains crucial to filter false positives and confirm SSI cases definitively.

Several factors may account for the lower specificity, including the complexity and variability in clinical documentation. Terms commonly associated with infections, such as “fluid collection,” or “wound drainage,” led to overclassification. This linguistic bias could arise from the model’s training on large datasets, where specific terms frequently correlate with infections.^7^ The model also struggled to distinguish between preoperative infection context and postoperative outcomes, misclassifying patients who underwent surgery to treat an existing infection (e.g., discitis or osteomyelitis).

Our study has several limitations to consider. It was conducted at a single tertiary care institution, which may limit the generalizability of the findings; it is unclear whether the prompt design used in this study would also exhibit 100% sensitivity in other settings. Our focus was on the initial feasibility of using LLMs for SSI adjudication rather than fine-tuning the model for optimal performance. Future studies should aim to address these limitations by evaluating the model across multiple institutions and refining prompt engineering and LLM training processes. The workload-reduction of 66% may also be an overestimate, as our institution already employs an EHR-based note-filtering system, “Bugsy,” to identify potential SSIs for manual review. Future studies should prospectively aim to compare the performance of Bugsy’s existing filtering mechanism with that of the LLM tool described here.

In conclusion, LLM-based agents show promise in assisting with SSI adjudication, given their potential for perfect sensitivity in our study. Their relatively modest specificity, however, showcases an ongoing need for human involvement in the process, and that the primary role for LLM in this context would be workload-reduction.

## Supporting information

10.1017/ice.2026.10432.sm001 Miranti et al. supplementary material Miranti et al. supplementary material

## Supplementary material

The supplementary material for this article can be found at https://doi.org/10.1017/ice.2026.10432.

## Figure 1 Long description

The flow diagram depicts the process of evaluating surgical site infections using a large language model. It begins with a prompt instructing the model to act as a medical language assistant to determine if a patient experienced a surgical site infection by reviewing clinical notes. The diagram includes the National Healthcare Safety Network (NHSN) logo, indicating the guidelines used for surveillance. All notes within the post-surgical 30- or 90-day observation period, depending on the surgery type, are reviewed. The model flags notes that may mention a surgical site infection for human review. If a patient has any flagged notes during their observation period, the patient is flagged for human review. The metrics for all surgical cases from 2023 are provided, with a sensitivity of 1.0 and a specificity of 0.694.

Navigate back to Figure 1.
